# High prevalence of high-risk HPV genotypes other than 16 and 18 in cervical cancers of Curaçao: implications for choice of prophylactic HPV vaccine

**DOI:** 10.1136/sextrans-2017-053109

**Published:** 2017-10-11

**Authors:** Desiree J Hooi, Birgit I Lissenberg-Witte, Maurits N C de Koning, Herbert M Pinedo, Gemma G Kenter, Chris JLM Meijer, Wim G Quint

**Affiliations:** 1 Fundashon Prevenshon, Willemstad, Curaçao; 2 Department of Pathology, VU University Medical Centre, Amsterdam, the Netherlands; 3 Department of Epidemiology and Biostatistics, VU University Medical Centre, Amsterdam, the Netherlands; 4 DDL Diagnostic Laboratory, Rijswijk, the Netherlands; 5 Department of Gynaecology and Oncology, VU University Medical Centre, Amsterdam, the Netherlands

**Keywords:** HPV prevalence, HPV genotypes, Cervical cancer, HPV vaccination, Cervical intraepithelial neoplasia(CIN), Curaçao

## Abstract

**Background:**

Curaçao is a Dutch-Caribbean Island located in a high-risk area for cervical cancer.

Prior to introduction of a prophylactic human papillomavirus (HPV) vaccine, knowledge of the prevalence of high-risk HPV vaccine genotypes (HPV16, 18, 31, 33, 45, 52 and 58) in cervical (pre)cancer is required.

**Objective:**

To investigate the prevalence of HPV genotypes in invasive cervical cancers (ICC) and cervical intraepithelial neoplasia (CIN) grade 1, 2 and 3 in Curaçao.

**Methods:**

Paraffin-embedded blocks of 104 cervical cancers (89 squamous, 15 adenocarcinoma), 41 CIN3, 39 CIN2 and 40 CIN1 lesions were analysed for the presence of HPV. Sections were stained by H&E for histopathological evaluation, and DNA was extracted using proteinase K. HPV genotypes were detected using Short PCR Fragment (SPF10) PCR DNA enzyme immunoassay and a Line Probe Assay (LiPA25).

**Results:**

HPV was found in 92 (88.5%) ICC; 87 (94.6%) had a single HPV infection and 86 (93.5%) were high-risk human papillomavirus (hrHPV)-type positive.

The three most common HPV types in ICC were 16 (38.5%), 18 (13.5%) and 45 (6.7%), covering 58.7%.

HrHPV vaccine genotypes 16, 18, 31, 35, 45, 52 and 58 were responsible for 73.1% of ICC. For precancerous lesions, the HPV attribution was 85.4% for CIN3, 66.7% for CIN2% and 42.5% for CIN1.

**Conclusions:**

Our study, the largest in the Caribbean region in (pre)cancer, shows that the prevalence of HPV-type 16 and 18 in cervical cancer is lower compared with the world population but no differences in prevalence of these two HPV types are seen in precancerous lesions.

When considering HPV vaccination in Curaçao, the relatively high contribution of non-HPV 16/18 genotypes in ICC should be taken into account.

## Introduction

Curaçao is a Dutch-Caribbean island with estimated 155 000 inhabitants last registered in 2014.^1^ It is demographically located in an area with a high incidence and mortality of cervical cancer^2–5^ known to be caused by high-risk HPV.^6 7^ According to The Cancer Registry of Curaçao, the incidence of cervical cancer over 2004–2008 is 13.4 per 100 000 women (CMD Coronel, personal communication, 2015).

The lack of an organised cervical screening and prophylactic HPV vaccination programme largely explains the high incidence of cervical cancer.^8–10^

Presently, three HPV prophylactic vaccines are available. A bivalent HPV vaccine against HPV16 and 18; a quadrivalent vaccine directed against HPV16 and 18 with an additional coverage to low-risk HPV 6 and 11; and recently a nonavalent vaccine directed against high-risk Human papillomavirus (hrHPV)16, 18, 31, 33, 45, 52 and 58 and lrHPV6 and 11. The bivalent and quadrivalent vaccines have shown partial to full cross-protection against certain non-vaccine HPV types.^11 12^

According to the report about the situational analysis of cervical cancer prevention and control in the Caribbean, only three Caribbean islands have a HPV vaccination programme up and running.^10^ In 2007, the first HPV vaccine was introduced on Curaçao. Both the bivalent and quadrivalent HPV vaccines are available and recently in 2017, the nonavalent HPV vaccine was introduced. These vaccines are not part of a National Vaccination Programme and are only available at their own expense. Family practitioners and paediatricians can prescribe the vaccines on patient’s request. At present, there are no standard HPV vaccination protocol or a national vaccination registry functioning. Less than a hundred vaccines were sold since the introduction in 2007, until resulting in a very low vaccine coverage (personal communication by S. Elhage, M. Ruijs and J. Boujon of the local vaccine agencies).

In July 2016, the island started with an HPV-based national cervical cancer screening programme.

Before introducing a prophylactic vaccine to women on Curaçao, it is important to know the prevalence of HPV genotypes in cervical precancerous lesions and cancer. This can help in the decision by policymakers which prophylactic HPV vaccine should be used and what the potential impact of the new vaccine might be. Here we describe the prevalence of HPV genotypes in paraffin-embedded tissues of cervical cancers and cervical precancerous lesions (cervical intraepithelial neoplasia grades 1, 2 and 3 (CIN1, CIN2, CIN3)) derived from the only pathology laboratory of Curaçao.

## Material and methods

### Material

A retrospective study was designed and co-ordinated by Fundashon Prevenshon (FP), Curaçao, in co-operation with DDL Diagnostic Laboratory, Rijswijk, the Netherlands; the department of pathology, VU University Medical Centre (VUmc) Amsterdam, the Netherlands and the Analytic Diagnostic Centre (ADC), Curaçao, to determine the prevalence of HPV DNA genotypes in formalin-fixed paraffin-embedded (FFPE) tissues of women with invasive cervical cancer (ICC).

All ICC and CIN cases diagnosed in woman from Curaçao were selected in the PALGA system, which is the nationwide network and registry of histopathology and cytopathology in the Netherlands and Curaçao. This system includes both private and government insured patients. Only available blocks of good quality, containing enough tissue, were selected from 2012 backwards, anonymised, relabelled and analysed at DDL diagnostic laboratory. In total, FFPE blocks of 104 cervical cancers, 40 CIN1, 39 CIN2 and 41 CIN3 were collected from the ADC’s department of Pathology, Curaçao. The type of tissue from which the lesion samples are obtained is given in table 1.

**Table 1:** Type of tissue sample of cervical cancer and CIN lesion used for HPV detection

	Cervical ca.	CIN3	CIN2	CIN1
Biopsies	75	30	27	25
Large loop excision of the transformation zone (LLETZ)	1	5	6	4
Intracervical curettages	6	4	3	2
Cervix conisations	14	1	2	8
Hysterectomies	8	0	1	2
Total	104	40	39	41

CIN, cervical intraepithelial neoplasia.

Eight sections were cut from each block according to the sandwich method for histopathology and DNA extraction for HPV testing.^7^

In brief, the first and last sections were 4 µm and stained with H&E for histopathological evaluation of the lesion. In between the two H&E-stained sections, six sections of 8 µm were cut. The first three were combined in a single tube and used for HPV genotyping, the last three were stored in another tube for back up.

After each 10 blocks that were cut, an empty paraffin block was cut as a control for contamination during the sectioning process. Sections from the control block were tested in parallel with the samples and remained negative. For each sample, we also obtained age of the woman at diagnosis, date of sample collection and histological diagnosis.

### DNA isolation and HPV testing

Tissue sections were incubated overnight for DNA extraction with 250 µL of a 0.1% proteinase K solution at 70ºC. The standard proteinase volume of 250 µL was reduced to 100 µL for sections containing small tissue samples as observed by visual inspection. After incubation, proteinase K was inactivated at 95ºC for 10 min. HPV DNA was amplified by the SPF10 PCR in a total volume of 50 µL using 10 µL of 10 times diluted DNA. The SPF10 DNA enzyme immunoassay (DEIA) (Labo Bio-medical Products, Rijswijk, The Netherlands) was used for general detection of over 69 known HPV types by hybridisation to a mixture of HPV-specific probes in a 96-well format.^13 14^

Amplimers from positive samples by DEIA were genotyped in the SPF10LiPA25 reverse hybridisation assay for identification of 25 HPV genotypes^15^ (version1; Labo Bio-medical Products, based on licensed Innogenetics technology).

DNA from all samples was also tested for the presence of amplifiable human genomic DNA and PCR inhibition by a duplex real-time PCR targeting the human RNaseP gene and a plasmid spiked into the PCR mix.^16^ In case the Ct value of the plasmid spike-in was elevated, indicating PCR inhibition, the already 10 times diluted sample was diluted 10 more times and re-tested for HPV and PCR inhibition. If the 10 times diluted sample did not show PCR inhibition and the HPV result was negative, the undiluted sample was re-tested for HPV and PCR inhibition.

### Statistical analyses

HPV type attribution per group was assessed in different ways to deal with multiple infections (see online supplementary table S1). First, we considered only single HPV infections. That is, women with multiple HPV infections were not included in the calculation, underestimating the genotype attributable fraction. Second, we considered each infection as a causal infection, that is, for a woman infected with HPV16 and HPV31, both 16 and 31 were counted as separate attribution, overestimating the genotype attributable fraction.^17^ Finally, we used hierarchical and proportional attribution to account for multiple HPV infections.^18^ In short, in a woman with multiple infections, either the HPV infection with the highest prevalence within the population (hierarchical method) or each HPV infection proportional to the total prevalence within the population (proportional method) is assumed to have attributed to the lesion.

10.1136/sextrans-2017-053109.supp1 Supplementary Table 1

Ethical approval of the study was obtained from the institutional review board (IRB) of the medical ethics committee of FP, Curaçao (IRB board’s approval number 0001/14).

## Results

Table 2 presents the mean age at which cervical cancer and CIN lesions were diagnosed, and the prevalence of HPV in these lesions.

**Table 2:** The prevalence of HPV, single HPV and multiple HPV infections and hrHPV in cervical cancer and CIN lesions

	N	Age (mean)	Age (range)	HPV positive	%	HPV negative	%	HPV single	%	HPV multiple	%	hrHPV* pos.	%
CIN1	40	32.7	(20–51)	31	77.5	9	22.5	21	67.7	10	32.3	24	77.4
CIN2	39	34.4	(19–72)	35	89.7	4	10.3	27	77.1	8	22.9	34	97.1
CIN3	41	38.6	(25–74)	39	95.1	2	4.9	31	79.5	8	20.5	39	100.0
Cancer (ICC)	104	52.6	(27–86)	92	88.5	3	20.0	87	94.6	5	5.4	86	93.5
SCC+ASC	89	52.2	(27–86)	80	89.9	9	10.1	75	93.8	5	6.3	74	92.5
ADC	15	51.9	(32–74)	12	80.0	12	11.5	12	100.0	0	0	12	100.0

*Percentage of hrHPV is calculated from the number of HPV-positive cases.

ADC, adenocarcinomae; ASC, adenosquamous carcinoma; CIN, cervical intraepithelial neoplasia; ICC, invasive cervical cancer; SCC, squamous cell carcinoma.

Since the results of hierarchical attribution analysis were similar to the proportional attribution analysis, we only present here the hierarchical distribution. The prevalence of HPV genotypes in multiple infections in cervical cancer and CIN lesions is shown in online supplementary table S1.

### Carcinomas

Most of the histotypes were squamous cell carcinoma (SCC n=85 (81.7%)), followed by adenocarcinoma ((ADC n=15 (14.4%)) and adenosquamous carcinoma ((ASC n=4 (3.8%)) (table 2).

Because of the small number of ASC, for statistical analysis we added the four ASC cases to the SCC.

The large majority (88.5%) of cervical carcinoma were HPV positive of which only 5.4% had a multiple infection. The majority of positive ICC cases (93.5%) contained one hrHPV type (table 2). The most prevalent HPV types in ICC were 16 (38.5%), 18 (13.5%) and 45 (6.7%), covering 58.7% of the carcinomas (table 3). Four out of the five multiple hrHPV infections in cervical cancer had HPV16 in combination with another high-risk type (see online supplementary table S2).

10.1136/sextrans-2017-053109.supp2 Supplementary Table 2

**Table 3:** HPV-type distribution in HPV-positive cervical cancer, subdivided into SCC/ASC, ADC and CIN 3, 2, 1 lesions.

Type	Hierarchical attribution*													
	Cancer		SCC/ASC		ADC		CIN3		CIN2		CIN1		Total	
	*n*	*%*	*n*	*%*	*n*	*%*	*n*	*%*	*n*	*%*	*n*	*%*	*n*	*%*
**hrHPV**														
**16**	40	38.5	36	40.4	4	26.7	19	46.3	12	30.8	6	15.0	77	34.4
**18**	14	13.5	8	9.0	6	40.0	3	7.3	4	10.3	1	2.5	22	9.8
**31**	4	3.8	4	4.5			5	12.2	2	5.1	5	12.5	16	7.1
**33**	3	2.9	3	3.4			1	2.4	2	5.1	1	2.5	7	3.1
**35**	3	2.9	3	3.4					4	10.3			7	3.1
**39**	1	1.0	1	1.1					1	2.6			2	0.9
**45**	7	6.7	5	5.6	2	13.3	2	4.9	1	2.6	2	5.0	12	5.4
**51**	3	2.9	3	3.4			2	4.9	4	10.3	3	7.5	12	5.4
**52**	4	3.8	4	4.5			3	7.3	2	5.1	1	2.5	10	4.5
**56**											1	2.5	1	0.4
**58**	4	3.8	4	4.5			3	7.3	1	2.6	2	5.0	10	4.5
**59**	1	1.0	1	1.1							1	2.5	2	0.9
**66**							1	2.4	1	2.6			2	0.9
**68**	2	1.9	2	2.2							1	2.5	3	1.3
**lrHPV**														
**6**									1	2.6	2	5.0	3	1.3
**44**														
**53**											1	2.5	1	0.4
**54**														
**61**											1	2.5	1	0.4
**67**	1	1.0	1	1.1									1	0.4
**70**											2	5.0	2	0.9
**71**														
**74**														
**82**	1	1.0	1	1.1									1	0.4
**91**														
**X**	4	3.8	4	4.5							1	2.5	5	2.2
**Total**	92	88.5	80	89.9	12	80.0	39	95.1	35	89.7	31	77.5	197	87.9
**Negative**	12	11.5	9	10.1	3	20.%	2	4.9	4	10.3	9	22.5	27	12.1

ADC, adenocarcinomae; ASC, adenosquamous carcinoma; CIN, cervical intraepithelial neoplasia; SCC, squamous cell carcinoma.

Eighty per cent of the ADC (n=15) were HPV positive.

Only single HPV-type infections were detected, and all samples were high-risk HPV types. HPV18 was the most prevalent HPV type (40.0%) followed by HPV16 (26.7%) and HPV45 (13.3%) (table 3).

In total, 12 of the ICC samples were HPV negative: nine SCC and three ADC.

### CIN lesions

The percentages of HPV prevalence increased with the grade of CIN lesion namely CIN1 77.5%, CIN2 89.7%, CIN3 95.1% (table 2). The percentages of multiple HPV infections decreased from CIN1 (32.3%) to CIN3 (20.5%)and were lowest in cervical carcinomas (5.4%).

HPV16 was the most prevalent HPV type in all degrees of CIN and its prevalence increased substantially with greater severity of CIN. The most prevalent HPV types in CIN3 samples were HPV16 (46.3%) and HPV 31 (12.2%). HPV types 18, 52 and 58 each covered 7.3%.

In CIN 2, the most common type was HPV16 (30.8%), while HPV18, 35 and 51 were the second most common, each covered 10.3%. In CIN 1, the most common HPV types were HPV16 (15.0%), HPV31 (12.5%) and HPV51 (7.5%) (table 3).

## Discussion

This is the first study from Curaçao to show systematic data on HPV prevalence in cervical cancer and CIN lesions. Interestingly, although the prevalences of HPV16 (38.5%) and HPV18 (13.5%) in cervical cancer of women from Curaçao are still the most prevalent HPV genotypes, their prevalence is lower compared with the world prevalence.^19–21^

The prevalence of vaccine types HPV31, HPV52, HPV58 and HPV X (DEIA pos. and LiPA neg.) for each HPV type was 3.8%, in total 15.2%. In ADC (n=15), HPV 18 (40.0%), HPV16 (26.7%) and HPV45 (13.3%) were the most prevalent HPV types. Twelve cervical cancers were found to be HPV negative.

We performed several experiments to substantiate absence of HPV in cervical cancer and exclude experimental causes. Review of the slides by two pathologists did not change the diagnosis, and repeated HPV testing and genotyping with SPF10 yielded identical results. Inadequate DNA quality could be excluded by parallel testing with a duplex real-time PCR targeting the human RNaseP gene. Potential PCR inhibition could be excluded by a plasmid spiked into the PCR mix.

All 12 samples contained amplifiable human genomic DNA and stable Cq values of the plasmid spike showed absence of PCR inhibition. Another explanation for a negative HPV result in cervical carcinoma might be integration of HPV DNA in the L1 region because the SPF10 targets the L1 gene. We did not perform HPV detection assays targeting the E6/E7 region to exclude this possibility.^22^ However, published data show that <1% of cervical cancers has integration in the L1 region.^23 24^

Our HPV 16/18 prevalence data in cervical cancer are in agreement with Martinique (57% in 131 ICC)^25^ but differ from those in Trinidad (83.9%).^26^

The ranking order of HPV genotypes found in cervical cancer in Curaçao matches with those from Trinidad HPV16 (66.1%), HPV18 (17.8%) and HPV45 (8.9%).^26^ Comparison with HPV genotype studies from Jamaica was difficult due to pooling of the small sample size (9 ICC and 30 CIN3 lesions).^27^

Compared with HPV 16 and 18 in cervical cancer from the Caribbean region according to the Institut Català d’Oncologia (ICO) data (57.6%), our estimated HPV16 and 18 prevalence data in cervical cancer are somewhat lower (52.0%), but our HPV 16 and 18 prevalence data in high-grade lesions (CIN2/3; 47.6%) are higher compared with the ICO data (CIN2/3; 34.6%).^19^

However, it should be noted that most of the published HPV prevalence data from CIN lesions in the Caribbean region are obtained from studies in women with normal cytology where these lesions were discovered incidentally during the trials. In Jamaica (n=840), the prevalence of HPV16 and 18 was found to be 50% in 14 high-grade squamous intraepithelial lesion (HSIL) and 21.3% in 61 atypical squamous cells of undetermined significance (ASC-US) and low-grade squamous intraepithelial lesion (LSIL).^28^

Data from Guadeloupe (n=618) only described HPV 16 and 18 prevalence in LSIL: 9.7% in LSIL (n=31) and 11.3% of ASC-US (n=106). HPV 16 and 18 prevalence in HSIL were not described because of the small sample size (n=8).^29^

An explanation for the differences in HPV prevalence between the Caribbean region may be the very heterogeneous ethnical background, which consists of a mix of African and European descent and different social and cultural behaviours in this population.

Our HPV genotype prevalence data argue for the use of a prophylactic HPV vaccine with broad coverage of the non-HPV 16/18 genotypes, as present in the nonavalent vaccine. The use of HPV vaccines with a smaller HPV genotype coverage such as the bivalent vaccine can also be considered when longitudinal data show that the cross-protection of the HPV 16/18 vaccines provides also long-lasting protection against non-vaccine HPV types such as 45, 31 and 33, present in relevant percentages in the population.^30 31^

The strengths of our study is that all samples derived from the only Pathology laboratory in Curaçao where all cases were handled according to one protocol. Three pathologists did histological review and HPV typing was carried out following a single standardised protocol in DDL, which has ample experience with HPV genotyping in very large epidemiological studies on genotyping.^20^

A limitation of the study is the limited number of CIN lesions studied because only available blocks of good quality and containing enough tissue were included. Consequently, this should be taken into account when interpreting the HPV prevalence data. However, this is the largest series of CIN from the Caribbean area in which HPV prevalence was assessed.

In conclusion, our study shows that compared with the world population the prevalence of HPV types 16 and 18 in cervical cancer is lower and consequently the contribution of the HPV genotypes 31, 45, 51, 52 and 58 is higher. When considering prophylactic HPV vaccination on Curaçao, these HPV genotypes' prevalence data in cervical (pre)cancer should be taken into account.

**Key messages**

- The incidence of cervical cancer in Curaçao is high (13.1) and cervical cancer prevention programmes are lacking.
- The prevalence of HPV16 and 18 in cervical cancer in this large study from Curaҫao is lower compared with the world population and Trinidad.
- The prevalence of HPV genotypes in CIN lesions in Curaҫao is comparable with the world population.
- In the selection of a prophylactic HPV vaccine the high proportion of non-HPV 16/18 genotypes in cervical cancer should be taken into account.

10.1136/sextrans-2017-053109.supp3 Supplementary file 1
