# Supplementary material for: High prevalence of high-risk HPV genotypes other than 16 and 18 in cervical cancers of Curaçao: implications for choice of prophylactic HPV vaccine
Source: Sex Transm Infect. 2017 Oct 11;94(4):263–7. doi: 10.1136/sextrans-2017-053109 (PMC5969325; doi:10.1136/sextrans-2017-053109)
Supplement: Supplementary Table 1 [file sextrans-2017-053109supp001.docx]

Supplementary table 1. HPV type attribution in relation to cervical (pre) cancer


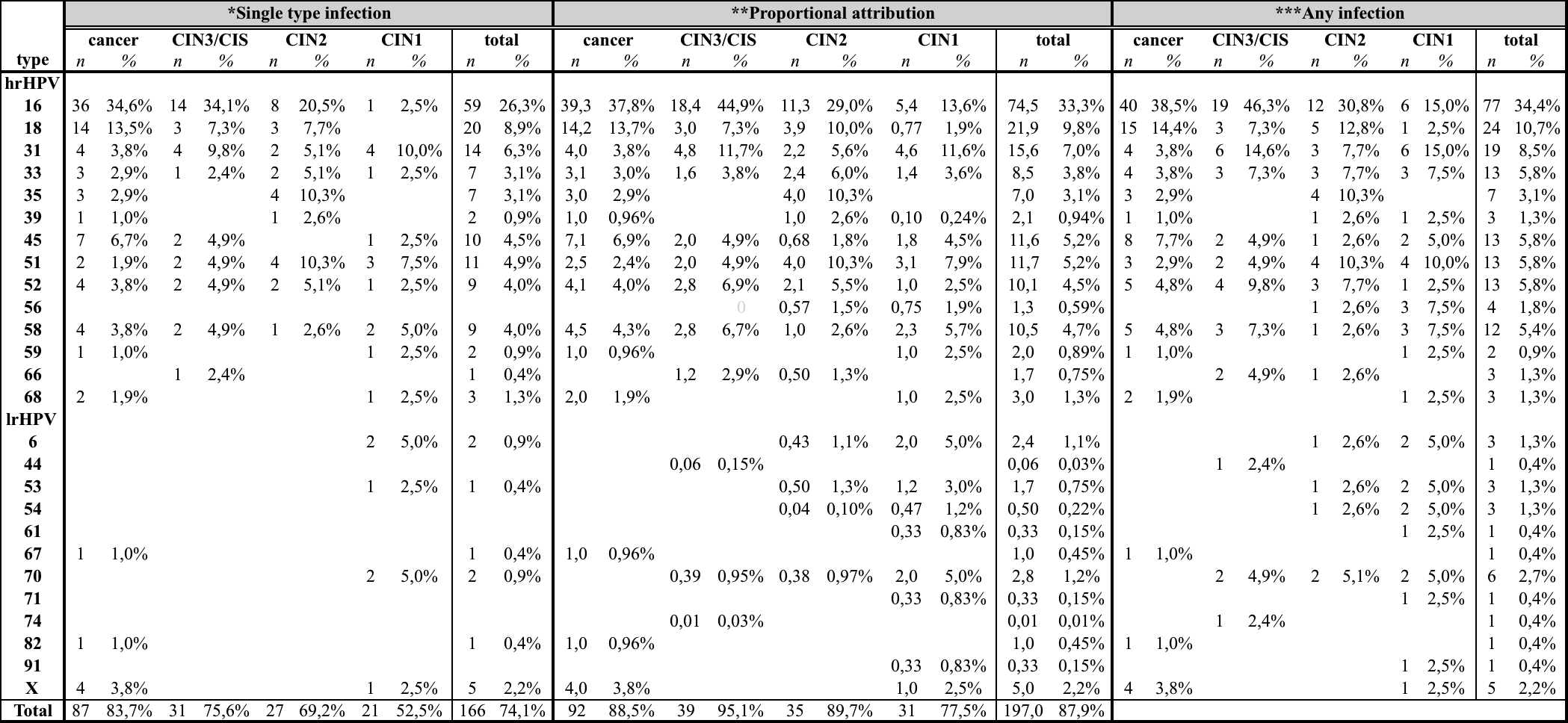


* ”Single type infection” is calculated by the frequency of each HPV genotype.

** ”Proportional attribution” of a specific HPV genotype in a multiple infection is assessed according to the frequency of that genotype at the respective disease category. (Wentzensen et al. 2010)

*** In ”any infection attribution” each infection is accounted fully to the lesion. (Wentzensen et al. 2010)
