# Supplementary figures and images for: High prevalence of high-risk HPV genotypes other than 16 and 18 in cervical cancers of Curaçao: implications for choice of prophylactic HPV vaccine
Source: Sex Transm Infect. 2017 Oct 11;94(4):263–7. doi: 10.1136/sextrans-2017-053109 (PMC5969325; doi:10.1136/sextrans-2017-053109)

Supplementary table 2. HPV genotypes in multiple HPV infections in cervical (pre)cancer.


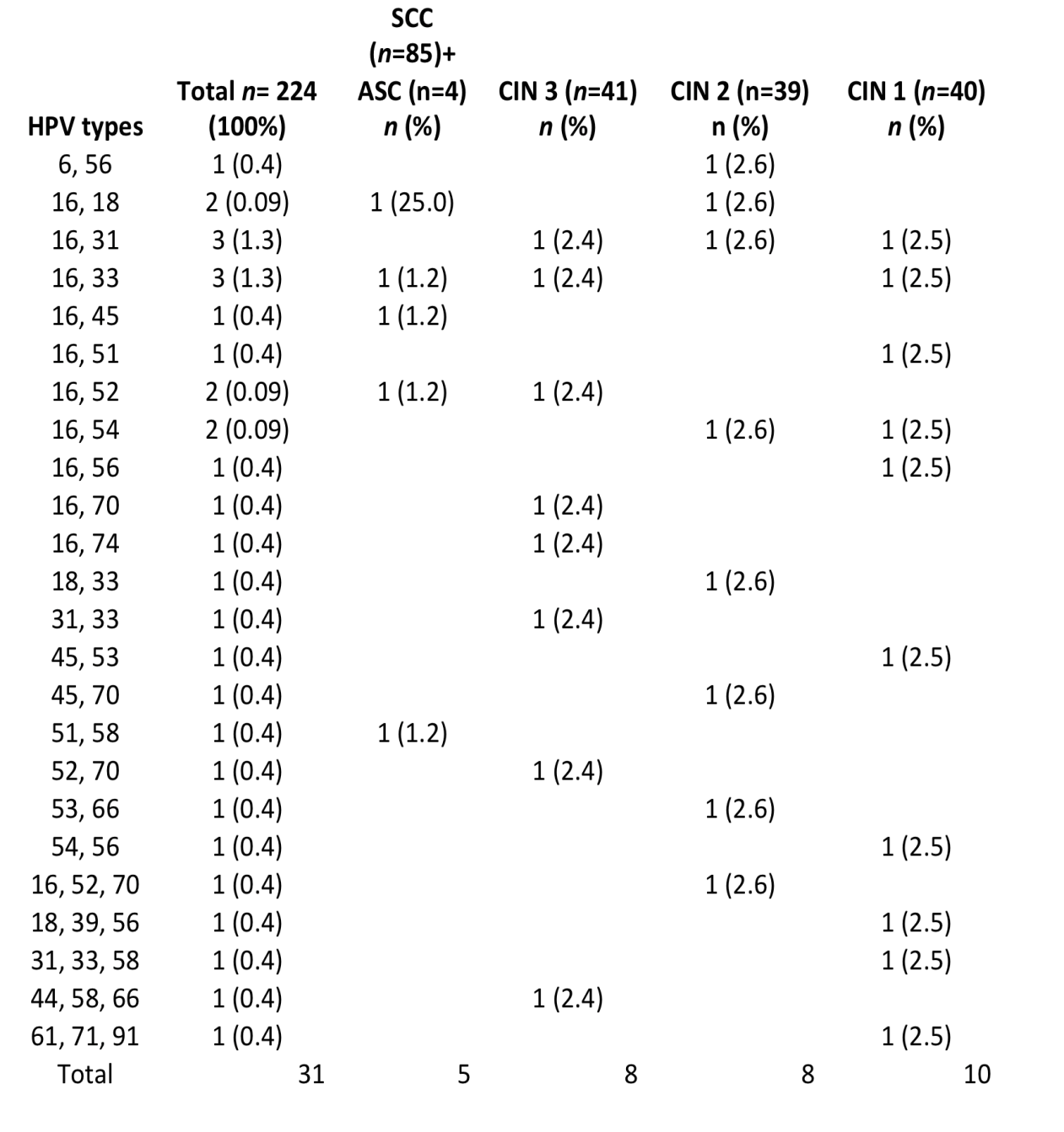

Supplement: Supplementary Table 2 [file sextrans-2017-053109supp002.docx]
