# Supplementary material for: High prevalence of high-risk HPV genotypes other than 16 and 18 in cervical cancers of Curaçao: implications for choice of prophylactic HPV vaccine
Source: Sex Transm Infect. 2017 Oct 11;94(4):263–7. doi: 10.1136/sextrans-2017-053109 (PMC5969325; doi:10.1136/sextrans-2017-053109)
Supplement: Supplementary file 1 [file sextrans-2017-053109supp003.pdf]

Prevalensia haltu di genotipo di riesgo haltu di VPH otro for di 16 i 18 den kanser na boka di matris na Kòrsou: implikashon pa ku eskoho di vakuna preventivo kontra di VPH

1. Fundashon Prevenshon, Kòrsou
2. DDL Diagnostic Laboratory, Rijswijk, Hulanda
3. VU Sentro Médiko Universitario, Departamentu di Epidemiologia i Bioestadistika, Hulanda
4. VU Sentro Médiko Universitario, Departamentu di Ginekologia i Onkologia, Hulanda
5. VU Sentro Médiko Universitario, Departamentu di Patologia, Hulanda

Palabranan klave: Prevalensia di VPH, genotipo di VPH, Kanser na boka di matris, vakuna di VPH, Leshon pre-kanseroso, Kòrsou
*VPH= Viro di Papiloma Humano

Introdukshon: Kòrsou ta un isla parti di Reino Hulandes den Karibe, hubiká den un zona ku riesgo haltu pa kanser na boka di matris.
Promé ku introdusí un vakuna preventivo kontra Viro di Papiloma humano (VPH), ta importante pa sa e prevalensia den leshonnan pre-kanseroso i kanser, di e tipo di VPH di riesgo haltu (VPH 16, -18, -31, -33, -45, -52, i -58) ku e vakuna kontra VPH ta kubri.
Meta: Investigá e prevalensia di genotipo di VPH den kanser invasivo i leshonnan pre-kanseroso grado 1,2 i 3 na Kòrsou.
Método: Hasiendo uzo di blòki di parafina, shentikuater kanser na boka di matris (89 squamous, 15 adenocarcinoma), 41 leshon pre-kanseroso grado 3, 39 grado 2, i 40 grado 1 a ser prosesá i analisá pa detektá presensia di VPH. Algun pida a ser kortá i despues prosesá ku Haematoxylin Eosin pa evaluashon histopatologiko i DNA a wòrdu ekstrahé pa medio di Proteinasa K. A hasi uso di e tèst: SPF10 PCR DEIA & LiPA25, pa detekshon di VPH i genotiponan spesífiko.
Resultado: VPH a ser detektá den 92 (88.5%) kanser invasivo na boka di matris; 87 (94.6%) tabata infektá ku solamente un di VPH i 86 (93.5%) tabata positivo pa un tipo di VPH di riesgo haltu. E tres tiponan di VPH mas komun detektá den kanser invasivo na boka di matris tabata 16 (38.5%), 18 (13.5%) i 45 (6.7%), na tur sumando 58.7%.
E genotiponan di VPH di riesgo haltu (ku e vakuna ta kubri) 16, 18, 31, 35, 45, 52, 58 tabata responsabel pa 73.1% di kanser invasivo na boka di matris. Den kaso di leshonnan pre-kanseroso, e kontribushon di VPH tabata repartí na e siguiente forma: 85.4% den grado 3, 66.7% den grado 2 i 42.5% den grado 1.
Konklushon: Nos investigashon esun di mas grandi den (pré)kanser pa ku e region di Karibe, ta mustra ku e prevalensia di VPH-tipo 16 i 18 den kanser na boka di matris ta tiki kompará ku otro poblashonnan na nivel mundial. Tambe e ta indiká ku no tabata tin diferensia entre poblashonnan den e prevalensia di e dos tiponan di VPH den leshonnan pre-kanseroso.
Na momentu di konsiderá un vakuna kontra VPH pa Kòrsou, mester tene na kuenta e kontribushon relativamente haltu di e tiponan di VPH ku no ta 16/18.

Alta prevalencia de genotipos de VPH de alto riesgo distintos a 16 y 18 en cánceres cervicales en Curaçao: implicaciones para la elección de la vacuna profiláctica contra el VPH

1. Fundashon Prevenshon, Curaçao
2. DDL Diagnostic Laboratory, Rijswijk, Países Bajos
3. VU University, Centro Medico Universitario, Departamento de Epidemiología y Bioestadística, Países Bajos
4. VU University, Centro Medico Universitario, Departamento de Ginecología y Oncología, Países Bajos
5. VU University, Centro Medico Universitario, Departamento de Patología, Países Bajos

Palabras clave: prevalencia del VPH, genotipos del VPH, cáncer cervical, vacunación contra el VPH, neoplasia intraepitelial cervical (NIC), Curaçao

*VPH= Viro de Papiloma Humano

Introducción: Curaçao es una isla en el Caribe, territorio Holandesa, ubicada en una zona de alto riesgo para el cáncer cervical. Previo la introducción de una vacuna profiláctica contra el Viro de Papiloma Humano (VPH), se requiere conocimiento de la prevalencia de genotipos de vacuna contra el VPH de alto riesgo (HPV16, -18, -31, -33, -45, -52 y -58) en el cáncer cervical .
Objetivo: Investigar la prevalencia de genotipos de VPH en cáncer cervical invasivo (CCI) y neoplasia intraepitelial cervical (NIC) grado 1, 2 y 3 en Curaçao.
Método: Se analizaron bloques de 104 cánceres cervicales (89 escamosos, 15 adenocarcinomas), 41 NIC3, 39 NIC2 y 40 NIC1 para la presencia de VPH. Las secciones fueron teñidas con Haematoxilina Eosina para la evaluación histopatológica y el ADN se extrajo con Proteínasa K. VPH-genotipos fueron detectados utilizando SPF10 PCR DEIA & LiPA25-ensayo.
Resultados: Se detectó VPH en 92 (88,5%) CCI; 87 (94,6%) tuvieron una sola infección por VPH y 86 (93,5%) fueron de tipo VPH de alto riesgo positivo.
Los tres tipos de VPH más comunes en ICC fueron 16 (38,5%), 18 (13,5%) y 45 (6,7%), con un 58,7%. Los genotipos de la vacuna VPH de alto riesgo 16, 18, 31, 35, 45, 52, 58 fueron responsables del 73,1% de ICC. Para las lesiones precancerosas, la atribución del VPH fue de 85,4% para NIC3, 66,7% para NIC2 y 42,5% para NIC1.
Conclusión: Nuestro estudio, la más extensa en (pre)cáncer en la región del Caribe, demuestra que la prevalencia de VPH tipo 16 y 18 en el cáncer de cuello uterino es menor en comparación con la población mundial, pero no se observan diferencias en la prevalencia de estos dos tipos de VPH en lesiones precancerosas. Al considerar la vacunación contra el VPH en Curaçao, se debe tener en cuenta la contribución relativamente alta de los VPH genotipos no- 16/18 en la CCI.

Hoge prevalentie van hoog risico HPV-genotypen anders dan 16 en 18 in baarmoederhalskanker op Curaçao: implicaties voor de keuze van profylactische HPV-vaccin

1. Fundashon Prevenshon, Curaçao
2. DDL Diagnostisch Laboratorium, Rijswijk, Nederland
3. VU Medisch Centrum, Departement Epidemiologie en Biostatistiek, Nederland
4. VU Medisch Centrum, Departement Gynaecologie en Oncologie, Nederland
5. VU Medisch Centrum, Afdeling Pathologie, Nederland

Trefwoorden: HPV-prevalentie, HPV-genotypen, Baarmoederhalskanker, HPV-vaccinatie, Cervicale intraepitheliale neoplasie (CIN), Curaçao

*HPV= Humaan papillomavirus

Achtergrond: Curaçao is een Nederlands-Caribisch eiland gelegen in een risicogebied voor baarmoederhalskanker.
Voorafgaand aan de introductie van een profylactisch Humaan papillomavirus (HPV)-vaccin is kennis van de prevalentie van HPV-vaccingenotypen met hoge risico's (HPV16, -18, -31, -33, -45, -52 en -58) in cervicale (voor) kanker vereist.
Doelstelling: Het onderzoeken van de prevalentie van HPV-genotypen in invasieve baarmoederhalskanker (ICC) en cervicale intraepitheliale neoplasia (CIN), 1, 2 en 3 in Curaçao.
Methoden: Paraffine blokken van 104 baarmoederhalskanker (89 plaveisel-, 15 adenocarcinoom), 41 CIN3, 39 CIN2 en 40 CIN1 laesies werden geanalyseerd voor de aanwezigheid van HPV.
Secties werden gekleurd door Hematoxyline-Eosine voor histopathologische evaluatie en DNA werd geëxtraheerd onder toepassing van Proteïnase K. HPV-genotypen werden gedetecteerd onder toepassing van SPF10 PCR DEIA & LiPA25-assay.
Resultaten: HPV werd gevonden in 92 (88,5%) ICC; 87 (94,6%) had een enkele HPV-infectie en 86 (93,5%) waren hrHPV-type positief.
De drie meest voorkomende HPV types in ICC waren 16 (38,5%), 18 (13,5%) en 45 (6,7%), die 58,7% bedroegen.
HrHPV-vaccingenotypen 16, 18, 31, 35, 45, 52, 58 waren verantwoordelijk voor 73,1% ICC.
Voor pre-kankerlaesies was de HPV-toeschrijving 85,4% voor CIN3, 66,7% voor CIN2 en 42,5% voor CIN1.
Conclusies: Onze studie, het grootste op (pre)kanker weefsel in de Caribische regio, laat zien dat de prevalentie van HPV-type 16 en 18 bij baarmoederhalskanker lager is dan de wereldbevolking, maar er zijn geen verschillen in prevalentie van deze twee HPV-typen in 'prekanker laesies'.
Bij het overwegen van HPV-vaccinatie op Curaçao, moet rekening worden gehouden met de relatief hoge bijdrage van niet-HPV 16/18 genotypen in ICC.

This abstract has been translated and adapted from the original English-language content. Translated content is provided on an "as is" basis. Translation accuracy or reliability is not guaranteed or implied. BMJ is not responsible for any errors and omissions arising from translation to the fullest extent permitted by law, BMJ shall not incur any liability, including without limitation, liability for damages, arising from the translated text.
